# Expanding Participation in Inclusive Physical Education: A Maker-Based Approach for Sport-Marginalized Students

**DOI:** 10.3390/children12121681

**Published:** 2025-12-10

**Authors:** Yongchul Kwon, Donghyun Kim, Minseo Kang, Gunsang Cho

**Affiliations:** Department of Physical Education, Pusan National University, Busan 46241, Republic of Korea; kyc6141@pusan.ac.kr (Y.K.); minseo1035@pusan.ac.kr (M.K.)

**Keywords:** marginalized students, maker education, inclusive education, physical education, professional learning communities, teacher professional development

## Abstract

**Highlights:**

**What are the main findings?**

• Three distinct maker-based lesson types were developed: (1) physical data measurement and analysis, (2) performance feedback, and (3) play- and game-based activities. These lessons expanded participation among sport-marginalized students by offering alternative and meaningful roles.
• Teachers reported shifts in their perceptions of inclusion, which led to instructional redesign and reconfiguration of their professional identity.

**What is the implication of the main finding?**

• Maker-based physical education offers a collaborative and student-centered approach that supports inclusive participation and learning in school PE curricula.
• The study provides a practical framework for innovating physical education through teacher collaboration, enabling equitable and engaging experiences for all students, especially those marginalized in traditional sport settings.

**Abstract:**

**Background/Objectives:** This study examined how maker-based physical education (PE) lessons, co-designed within a Professional Learning Community (PLC), expanded student participation and supported teacher professional growth. Focus was placed on engaging sport-marginalized students, often excluded due to ability, motivation, or social background. **Methods:** This qualitative single-case study examined a PE-focused professional learning community (PLC) that collaboratively designed maker-based PE lesson prototypes and partially implemented them in regular PE classes. Data included PLC documents, lesson plans, classroom observations, student work, and semi-structured teacher interviews, and were analyzed using inductive category analysis. **Results:** Three lesson types emerged: (1) physical data measurement and analysis, (2) performance feedback, and (3) play- and game-based formats. These diversified participation by promoting student roles beyond performers, such as creators and analysts. Sport-marginalized students took on new roles as creators and analysts and, at the same time, showed increased engagement in physical activities and more active participation in lessons as performers. Teachers shifted from skill-focused instruction to reflective, practice-based teaching. The PLC enabled sustained innovation and collective growth. **Conclusions:** Maker-based PE offers a low-cost, adaptable model for inclusive curriculum reform that promotes creativity, wellbeing, and participation. Future studies should explore its long-term impact, broader implementation, and strategies to support ongoing PLC-based innovation.

## 1. Introduction

Contemporary education has increasingly shifted from transmission-based teaching toward pedagogical approaches that emphasize learner agency, creativity, and meaningful participation [1]. This shift calls for students to actively construct knowledge, engage in collaborative inquiry, and develop competencies through authentic experiences. Within this paradigm, physical education (PE) faces growing pressure to move beyond traditional models centered on performance, competition, and standardization. However, despite ongoing reforms, many students continue to experience exclusion in PE contexts.

Sport-marginalized students are those whose participation is limited by factors such as physical ability, motivation, gender, disability, or socioeconomic background [2]. These students remain underserved in many PE programs. Such exclusionary patterns represent a global concern and highlight the urgent need for inclusive PE practices that provide equitable opportunities for engagement and achievement.

To address these disparities, pedagogical frameworks such as Cooperative Learning, the Sport Education Model, and Teaching Personal and Social Responsibility (TPSR) have been implemented in PE [3,4]. These models promote student-centered learning and collaborative interaction, offering partial pathways toward inclusion. In parallel, recent efforts to integrate STEAM education, project-based learning, and interdisciplinary approaches into PE have gained momentum [5]. Nevertheless, participation barriers remain persistent, particularly for students who do not identify with performance- or sport-based paradigms. This indicates a need for pedagogies that are more flexible, participatory, and creativity-oriented.

In this context, Maker Education has emerged as a promising alternative. As a practice-oriented pedagogy, Maker Education encourages learners to define problems, create solutions, and build artifacts using both digital and physical tools [6,7]. It fosters autonomy, problem-solving, and creativity while supporting diverse forms of participation. Research indicates that maker-based environments enhance engagement and self-efficacy among students who are often marginalized in traditional classroom settings, including those from disadvantaged socioeconomic backgrounds, girls, and students with disabilities [8,9,10]. Recent reviews also show a rapid expansion of K–12 maker education research, school-based implementation projects, and teacher training initiatives, highlighting the growing significance of maker pedagogy in formal education contexts [11,12,13].

The potential of Maker Education to promote inclusivity aligns well with the central goals of PE, particularly when participation is reimagined as creative, collaborative, and student-led. Despite this promise, empirical, subject-specific applications—especially in PE—remain scarce in the literature [14,15], indicating that maker-based pedagogy in physical education is still underexplored. Integrating maker principles into PE invites a pedagogical shift, enabling students to engage not only as performers but also as creators, operators, and analysts of physical activity, thereby broadening what it means to participate in PE.

At the international policy level, inclusive education advocates for all learners to have equitable access and meaningful participation in learning communities [16]. In PE, the persistent marginalization of certain student groups directly challenges these goals. Embedding Maker Education within PE offers a viable means to address this gap. By incorporating creativity, autonomy, and problem-solving into physical activity, this approach provides both practical and policy-relevant solutions.

To implement such innovations effectively, collaboration among teachers is essential, and professional learning communities (PLCs) have been identified as a key structure for sustaining professional development and innovation in PE [17,18,19]. In this study, the term Professional Learning Community (PLC) refers to an ongoing, collaborative group of educators who jointly inquire into their practice, design lessons, and reflect on student learning. Rather than a one-off workshop, a PLC operates as a sustained structure in which teachers regularly meet to share evidence from classrooms, co-design instructional strategies, and support one another’s professional growth. In the Korean context, PLCs are often organized as school- or district-level “teacher learning communities” supported by local education authorities, but the underlying idea is consistent with international understandings of PLCs as collaborative, inquiry-oriented professional groups.

While previous studies have examined inclusive pedagogies in PE, few have investigated how maker-based approaches can support sport-marginalized students. This study seeks to address that gap by examining how a PLC collaboratively designed and implemented maker-based PE lessons. Specifically, it aims (1) to identify the characteristics of lesson designs that enhance the participation of sport-marginalized students and (2) to explore how teachers’ collaborative reflections shaped their understandings of inclusivity.

By doing so, this study extends the theoretical and practical discourse on inclusive PE and offers insights into how Maker Education can reframe both student engagement and teacher professionalism in PE.

## 2. Research Design and Methods

### 2.1. Research Design

This study employed a qualitative embedded single-case design [20] to explore the types of PE lessons that integrated Maker Education and to examine their potential to broaden the participation of sport-marginalized students. The bounded case was a Professional Learning Community (PLC) that collaboratively designed and implemented lessons between 2023 and 2025. The PLC served as the single case, with two embedded units of analysis: (a) the characteristics of the implemented lesson types (RQ1) and (b) teachers’ perceptions and reflections (RQ2). This design is appropriate for producing rich, context-dependent descriptions of instructional phenomena and for generating practice-oriented implications relevant to school settings [21].

Data sources included PLC artifacts and records such as lesson plans, student work, and prototype development logs, as well as semi-structured interviews with five PE teachers. The data were analyzed using inductive category analysis to identify themes and classify the lesson types. Trustworthiness was enhanced through triangulation of data sources, peer debriefing, and researcher reflexivity.

This qualitative case study followed an iterative design-and-implementation approach: all maker-based lesson prototypes were piloted in actual PE classes, and the findings are based on empirical data generated during these implemented lessons and PLC activities.

### 2.2. Participants

This study involved five in-service PE teachers based in Busan, South Korea, who were members of a Maker Education Professional Learning Community (PLC) active between 2023 and 2025. All participants had previously taken part in a researcher-led maker-based lesson study and had collaboratively implemented a laser-shooting program that integrated Maker Education into PE. This prior experience provided a shared foundation for understanding maker-based pedagogy and informed the design of new lesson types aimed at supporting sport-marginalized students.

Participants were selected through purposive sampling, a method considered appropriate for qualitative case study research [22]. The group consisted of teachers with between five and twenty-five years of teaching experience at the middle and high school levels. All participants had received prior training in maker-based instructional practices through local education workshops and ongoing support via the PLC.

In the participating teachers’ classes, the maker-based PE lesson prototypes were implemented or piloted with students in middle and high school physical education classes, corresponding to the lower and upper secondary levels in the Korean education system. The students were typically between 13 and 18 years of age, which may facilitate comparison with secondary school contexts in other countries.

Before data collection, all teacher participants were fully informed about the study’s aims, procedures, and ethical considerations, and written informed consent was obtained.

### 2.3. Data Collection

Data were collected from three primary sources: PLC activity records, lesson implementation artifacts, and teacher interviews. First, documents generated during PLC activities, including meeting minutes, co-designed lesson plans, prototype development logs, and shared materials, were compiled to trace the evolution of lesson design and identify key challenges and collaborative responses.

Second, selected prototypes were piloted in PE classes. Observation notes, teacher reflective journals, and student artifacts (e.g., worksheets, photographs, short video clips) were collected to examine patterns of student participation and practical issues encountered during implementation.

Third, semi-structured interviews were conducted with the five participating teachers. Each teacher took part in one or two interview sessions, resulting in a total of 8 interviews. Each interview lasted approximately 60 min, was audio-recorded with participants’ consent, and subsequently transcribed verbatim. These interviews aimed to explore teachers’ instructional experiences and reflective insights regarding maker-based and inclusive PE practices.

Observation notes and teacher reflective journals included brief records of students’ in-class comments and visible reactions, which provided indirect but important insights into sport-marginalized students’ experiences.

### 2.4. Data Analysis

The data were analyzed using inductive category analysis. All PLC artifacts, lesson implementation materials, and interview transcripts were read in full to build an overall understanding of the case. Interviews were audio-recorded, transcribed verbatim, and organized in Microsoft Excel to enable flexible cross-referencing and iterative refinement of codes.

In the first coding cycle, 1st-order concepts were generated, remaining close to participants’ words and to specific incidents related to lesson design, the use of maker devices, and patterns of participation—particularly among sport-marginalized students. In the second cycle, related codes were grouped into broader 2nd-order themes, which were further refined into a small set of aggregate dimensions. This three-level structure helped to reveal core processes and patterns across the data.

To ensure the trustworthiness of the analysis, we employed researcher reflexivity, triangulated PLC documents, classroom artifacts, and interview data, and conducted peer debriefing with PE scholars. Coding decisions and category labels were reviewed collaboratively until agreement was reached, and the final categories were aligned with the research questions. Key findings are summarized in Table 1.

### 2.5. Researcher Positionality and Reflexivity

In this study, the researcher assumed multiple roles as designer, implementer, participant, and investigator. As a member of the PLC, the researcher was actively engaged in designing and delivering maker-based PE lessons, while also using the resulting lesson plans, worksheets, field notes, and student responses as data sources. This insider position provided valuable insight into the contextual dynamics and instructional processes.

However, such dual positioning posed a risk of interpretive bias, as the researcher’s involvement could influence both the generation and interpretation of data. To address this concern, the researcher maintained a reflexive journal throughout all stages of the study, critically examining personal assumptions and decision-making. Peer debriefing was conducted with PE colleagues, and data triangulation was used to enhance analytical credibility [23]. Transparent disclosure of the researcher’s role and its potential influence on the findings was upheld as a core methodological principle. By systematically acknowledging and addressing these risks, the study aimed to ensure methodological rigor and trustworthiness.

### 2.6. Ethical Considerations

All procedures adhered to established ethical guidelines for research involving human participants. Prior to data collection, participants were fully informed of the study’s purpose, procedures, data protection measures, the voluntary nature of their participation, and their right to withdraw at any time. Written informed consent was obtained from all participants.

Interviews were audio-recorded with participants’ permission, and all data were anonymized, treated confidentially, and used solely for research purposes. These safeguards were implemented to minimize potential risks and ensure the protection of participants throughout the research process.

## 3. Results

### 3.1. Maker-Based Lesson Types for Enhancing the Participation of Sport-Marginalized Students

Teachers within the PLC developed strategies to enhance the participation of sport-marginalized students in PE through the application of Maker Education. Through collaborative planning, implementation, and reflection, these strategies were translated into practice and categorized into distinct lesson types. The analysis identified three primary types: (1) Physical Data Measurement and Analysis, (2) Performance Feedback, and (3) Play- and Game-Based Lessons. Each type demonstrated potential to promote inclusivity in PE by offering marginalized students alternative and meaningful pathways for participation. The specific features of each lesson type are summarized in Table 2.

#### 3.1.1. Physical Data Measurement and Analysis Lessons

The PLC developed a lesson type in which students measured and analyzed their own physical data to increase the participation of sport-marginalized students. This approach addressed common challenges in school PE, such as limited equipment, manual data recording, and low student autonomy, while also providing new roles through which students could participate. Prototypes including a flexibility meter, height measurement tool, and heart rate monitor were created and tested. These tools supported students in recognizing and reflecting on their physical progress, which motivated them to participate more meaningfully in PE. Rather than simply recording results, students set goals and took ownership of their learning, particularly those who had previously remained passive.

The flexibility meter was designed to address the lack of motivation observed in traditional sit-and-reach tests, particularly among students with low motor ability. In most schools, only a single device was available, which restricted accessibility. The Professional Learning Community (PLC) developed a low-cost version that students could assemble and operate independently. This device was constructed using Arduino boards, which function as microcontroller platforms for physical computing, alongside TOF (Time of Flight) sensors for distance measurement and LCD screens for display. Test results were recorded and displayed instantaneously. Based on teachers’ observations and analysis of lesson artifacts, students demonstrated a greater sense of achievement, enhanced motivation for learning, and more active participation.

“In the past, we only had one flexibility tester at school, so students spent a lot of time just waiting in line, and the measurement itself often broke down. The students who were already flexible kept coming back to show off their scores, while those with low flexibility stayed away from the station altogether. When we used the new flexibility meter, however, even students with low flexibility started measuring their own scores and saying things like, ‘Let me try one more time to see if my number goes up.’ They were actively trying to improve their records.”

(Teacher D)

The height measurement device enabled students to monitor their growth regularly and independently. Traditional stadiometers are often bulky and not easily accessible. To address this issue, the PLC developed a compact version using a TOF sensor for distance measurement and an LCD screen for display. Students who were less inclined to participate in physical activities engaged with the device, becoming more aware of and interested in their physical development.

The heart rate monitor was designed to enhance students’ understanding of physiological responses to exercise. While many students were aware of changes in their heart rate, they often failed to connect these changes to the intensity of physical activity. A fingertip sensor with a small OLED display provided real-time heart rate data. This enabled students to monitor their bodily responses and supported the development of self-regulation. For marginalized students, the use of data rather than performance offered an alternative and meaningful avenue for engagement.

“For some students who usually avoid running, watching their heart rate numbers change became a trigger for greater curiosity and more active participation in the activity.”

(Teacher B)

**Figure 1:**
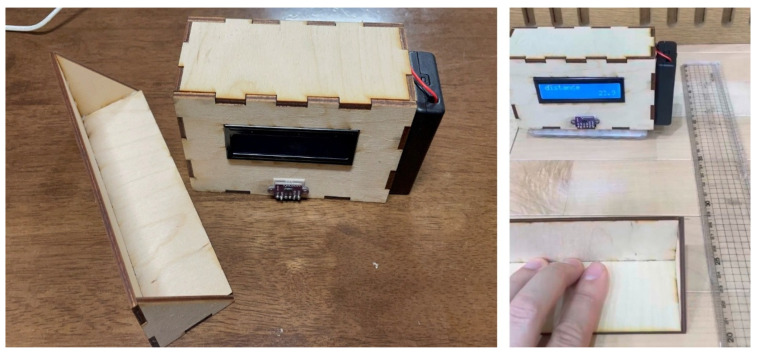
Prototype of the flexibility measurement device and its application in the classroom.

In summary, this lesson type enabled students to actively measure and analyze their physical data using self-constructed tools. It offered alternative roles and participation opportunities for marginalized students, thereby enhancing their engagement and inclusion in PE. Figure 1 presents the flexibility meter as a representative example.

#### 3.1.2. Performance Feedback Lessons

Traditional PE classes often used limited tools like measuring tapes and stopwatches, which reduced fairness and accuracy. Equipment shortages also restricted opportunities for repeated practice and discouraged participation among students with lower physical ability. To address these issues, the PLC developed performance feedback devices that students could design, build, and operate. Key examples included an automatic start sensor, a long jump measuring device, and a shuttle-run buzzer.

The automatic start sensor supported students who struggled with running tasks or felt discouraged during timed trials. In typical classes, timers were operated manually, which often reduced measurement accuracy. The PLC integrated Arduino with infrared and optical sensors to create a system that automatically started and stopped timing based on motion detection, providing more objective feedback and meaningful roles for marginalized students as device builders and users.

“When we used the automatic start sensor, students were excited that they could build the device themselves, and they were fascinated by how accurately it recorded their times after each run. Because they could check their results immediately, many of them kept saying, ‘Let me try one more time,’ and continued challenging themselves to improve their records.”

(Teacher E)

The long jump measuring device addressed the limitations of tape measures and subjective assessments by combining Arduino with laser distance sensors to display jump distances instantly on an LCD screen. This allowed students to monitor their progress through repeated practice. Marginalized students gained confidence by participating as both makers and users.

“With the long jump device, students could measure and check their distances right away, and seeing their numbers change over time encouraged them to keep trying to beat their previous records. One student who usually avoided the long jump because of low scores said that being able to measure himself without others watching made the activity feel much more comfortable.”

(Teacher A)

The shuttle-run buzzer was created to support students who were discouraged by low endurance or the pressure of performance-based evaluation. Using ultrasonic and TOF sensors, the buzzer signaled when a student reached the designated line, helping them maintain appropriate pacing. In trials, students modified the sound settings and added LED feedback, and they demonstrated self-directed behavior by independent planning and engaging in practice.

**Figure 2:**
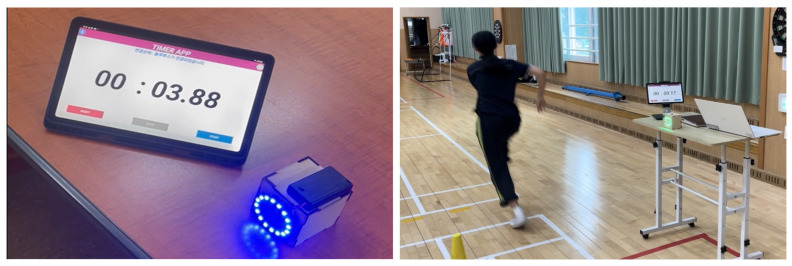
Prototype of the automatic timing start sensor and its classroom application. The Korean phrase on the screen means “Bluetooth is connected”.

Overall, performance feedback lessons helped students monitor and reflect on their exercise outcomes using self-made tools, promoting continuous learning. This approach reduced the limitations of competitive evaluation and opened new participation pathways for marginalized students. Figure 2 presents the automatic start sensor as a representative example.

#### 3.1.3. Play- and Game-Based Lessons

The PLC adapted previously developed measurement and feedback devices into play- and game-based lesson types to improve student participation and engagement. This redesign transformed assessment-focused lesson structures into playful and challenging experiences, encouraging sport-marginalized students to participate more actively in PE.

First, running games were created using buzzers and start sensors originally designed to record start and finish times. These were restructured into team competitions and cooperative activities, allowing students to move beyond record-based competition and work toward shared goals. Marginalized students assumed meaningful roles within these teams.

“Students who were usually marginalized in sport became highly engaged when we played games using the devices they had built themselves. Some proudly said, ‘This is the one I made,’ and took the lead in using it during the game.”

(Teacher B)

Second, cooperative fitness games employed shuttle-run buzzers and repetition-counting devices. These games awarded points when students met preset goals for repetitions or intensity. Real-time feedback and a sense of accomplishment kept even less physically skilled students motivated and engaged.

Third, laser shooting relay games were based on devices from the researcher’s previous study [14]. The PLC redesigned them as relay-style competitive games. Students constructed and used the shooting tools while participating in team-based scoring challenges, integrating design, operation, and performance. This process allowed marginalized students to shift from passive participants to active users and team contributors, enhancing both confidence and motivation.

“Another teacher reported, “In the laser shooting activities, it was often the usually quiet or hesitant students, rather than the most physically skilled, who achieved the best scores. When we combined shooting with relay races, all students, regardless of ability, joined in enthusiastically and said that the game felt both fun and fair.”

(Teacher D)

**Figure 3:**
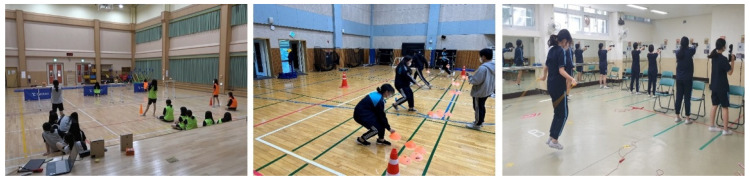
Classroom application of play- and game-based lesson.

Overall, these play- and game-based lessons offered marginalized students new ways to engage and succeed by incorporating gaming elements into feedback devices. Observations and PLC reflections showed that these students were more involved in game-based lessons than in traditional measurement-focused sessions. By turning routine assessments into enjoyable and cooperative experiences, this lesson type demonstrated strong potential to improve inclusivity in PE. Figure 3 presents an example of classroom implementation.

### 3.2. Teachers’ Reflections on Inclusivity Through Maker Education Practice

Based on teacher interviews and qualitative analysis, teachers reported that implementing Maker Education in PE diversified participation structures and opened alternative avenues for students marginalized by differences in physical ability. The analysis yielded three categories: (1) shifting perceptions of inclusivity and participation opportunities for marginalized students; (2) teachers’ professional reflection and instructional redesign; and (3) renewed recognition of the value of collaborative learning communities.

#### 3.2.1. Shift in Perceptions of Inclusivity and Participation Opportunities for Marginalized Students

Teachers reported that implementing Maker Education in PE diversified participation structures and opened alternative pathways for students previously marginalized due to differences in physical ability. Students were no longer confined to the role of “performers”; rather, they contributed as device makers, record users, and data analysts. Through these roles, they gained confidence and, in some cases, assumed central roles in class activities. For example, one student managed peers’ performance records, moving beyond the image of “not being good at PE,” while another actively supported classmates using equipment they had built.

In the past, students with lower scores gave up easily. When they used devices they had made themselves, they became the protagonists and were more motivated to participate in physical activity.

(Teacher A)

Students who were once regarded as not good at PE gained confidence and collaborated more actively with their peers when they took charge of making or operating devices.

(Teacher B)

Teachers also described a shift in their perceptions of marginalized students. Whereas learners with lower physical ability or confidence had often been regarded as “passive participants,” classroom episodes during making, operating, and analyzing activities revealed these students’ active engagement and contributions. This prompted teachers to reconceptualize them as capable and agentic learners.

“Even with lower physical ability, students were able to engage in meaningful learning by making devices and measuring performance. I realized that their limited participation reflected my own failure to recognize and understand their needs.”

(Teacher C)

In summary, across classroom observations and interviews, teachers affirmed that Maker Education enabled PE to move beyond performance-centered structures toward models that incorporated making, operating, and supporting roles. Through this process, marginalized students were redefined as active learners undertaking meaningful responsibilities, repeatedly reaffirming the inclusivity of PE.

#### 3.2.2. Teachers’ Professional Reflection and Instructional Reconfiguration

Teachers reported that engaging in Maker Education prompted them to reexamine their instructional routines and to explore new pedagogical strategies that integrate technology with PE. In the past, conventional PE was often standardized around physical activity and sport-specific skills. This emphasis led some teachers to hesitate when trying new approaches. However, the implementation of making activities allowed teachers to directly observe increases in students’ interest and engagement, as well as more diverse participation pathways. This shift went beyond minor methodological adjustments and encouraged reflection on teachers’ educational philosophies and the reconfiguration of their professional identities.

“I used to think PE was only about teaching movement, but once it was connected with technology, students’ engagement changed. I now understand why I must continue to explore diverse pedagogical approaches.”

(Teacher E)

“I realized that teachers also need to embrace new challenges. It’s not just about using equipment; the making process itself can be education.”

(Teacher B)

Some teachers also described a change in instructional focus. Previously, their attention tended to center on students with high motor proficiency or those expected to improve quickly. Through Maker Education, however, teachers recognized the need to design participation pathways specifically for students who had been marginalized in PE.

“When I focused solely on motor skills, my attention went mainly to students who performed well. Through Maker Education, I began to think about lessons for students who had been left out.”

(Teacher C)

These experiences showed that PE could be reframed from a focus on skill acquisition to a more integrative learning environment that combines technology, creativity, and collaboration. Working with students to solve technical issues during lessons also led teachers to see themselves as co-learners in the process. In some cases, they began drafting repeatable routines for future lessons, organizing lesson phases more clearly, informally documenting basic making procedures, and sharing strategies for assigning student roles with colleagues.

In summary, the practice of Maker Education gave teachers new perspectives on curriculum design and instructional methods. It supported the reconfiguration of professional identity in PE and helped foster multiple participation pathways for marginalized students. These changes laid the groundwork for shifting PE away from competition- and performance-centered models and toward more inclusive and collaborative approaches to teaching and learning.

#### 3.2.3. Re-Recognizing the Value of Collaboration and PLCs

Teachers consistently noted that the implementation of Maker Education could not be achieved through individual effort alone. Because equipment fabrication and classroom application required both technological and pedagogical understanding (for example, integrating sensor-based tools) and instructional management (for example, safety and the flow of activities), teachers reported considerable difficulty when working independently. Consequently, the Professional Learning Community (PLC) was regarded as an essential foundation for the practical implementation of maker-based PE.

During the project period, the PLC met regularly between May 2023 and August 2025. In total, ten face-to-face meetings were held, typically every 6–8 weeks for 90–120 min per session. These meetings were used to co-design maker-based PE lesson prototypes, review regular and pilot implementations, and reflect on students’ participation—particularly that of sport-marginalized students.

“Had I been alone, I would not even have attempted this. Working with colleagues who shared similar interests broadened my ideas and made implementation possible.”

(Teacher A)

Beyond simple information exchange, the PLC functioned as a practice-centered structure that made collaborative enactment possible and enhanced lesson feasibility. Meeting notes and shared files showed that teachers inspected circuit errors together, revised schematics, co-debugged timer malfunctions, uploaded code to a shared cloud folder, and standardized assembly procedures for sensors, LCDs, and batteries into step-by-step checklists for new participants. These collaborative outputs improved both lesson preparation efficiency and implementation accuracy.

“Thanks to the sample code shared in the PLC, problems that would have taken me days to fix on my own were solved within hours, especially by learning from others’ trial and error.”

(Teacher E)

A review of PLC minutes and post-workshop summaries confirmed that all participating teachers (*n* = 5) reported tangible benefits from sharing colleagues’ experiences and failures, such as preventing repeated mistakes, reducing preparation time, and accelerating precise problem solving.

In addition, the PLC served as a psychological support system that extended beyond technical and operational assistance. Teachers expressed less anxiety about trying new approaches alone and greater confidence in implementing maker-based lessons through collegial collaboration.

“The PLC was not just a research group but a source of mutual support and a driving force for creating new lessons. I was able to generate ideas to encourage marginalized students’ participation in physical activity and learn entirely new areas without undue burden.”

(Teacher D)

Collectively, the PLC fulfilled multiple functions, including technical troubleshooting, resource sharing and standardization, reduction in implementation burden, and psychological support. Through role division and reciprocal feedback, teachers enhanced the quality of lesson design and enactment. These experiences led them to reconceive the PLC not as a venue for training or information exchange, but as an action-oriented community of practice that reinforced the collective, practice-based professionalism that underpinned maker-based PE.

## 4. Discussion

This study was designed as a qualitative case study of a PE-focused Professional Learning Community (PLC) that engaged in the iterative design and classroom implementation of maker-based lesson prototypes. Rather than testing the effectiveness of a fixed intervention through standardized student outcome measures, the research aimed to generate in-depth, context-specific insights into how teachers co-constructed maker-based practices and how these practices opened up new participation pathways for sport-marginalized students. Accordingly, the discussion below focuses on interpreting the observed processes and patterns in light of existing theories of inclusive pedagogy and teacher agency, rather than claiming generalizable effects. These interpretations are grounded in classroom implementations rather than hypothetical lesson designs, as the PLC trialed each lesson type in regular PE classes and documented student participation and responses.

### 4.1. Expanding Inclusive Participation in PE Through Maker Practices

Maker-based PE provides an alternative instructional framework that moves beyond skill- and competition-centered lessons and enables more inclusive forms of participation. The findings indicate that Maker Education not only enhances engagement but also re-shapes how inclusion is realized in PE. While previous studies have typically addressed inclusion through rule modifications or task adaptations for students with lower physical abilities [24], this study shows that participation can be diversified through making, a process that integrates physical, cognitive, and technological engagement. This approach aligns with perspectives that emphasize agency through design in maker learning [25,26] and demonstrates how such practices can be effectively applied in PE contexts.

At the same time, the maker-based lessons in this study were not confined to cognitive or technical engagement with the devices themselves. The roles of “creator,” “operator,” and “analyst” were all embedded in embodied practice. For example, when students designed a running task using the automatic start sensor and installed the device, they then had to repeatedly perform the running task under the conditions they had created, adjust their speed and starting timing based on sensor feedback, and compare their own performance with that of their peers. Through these processes, the maker-based tasks provided structured opportunities for students with low motor competence to move beyond one-off performance and to rehearse and refine fundamental physical capacities—such as speed, strength, and rhythm—within a purposeful context.

These results can be interpreted through the lens of physical literacy [27], which emphasizes the integrated development of physical, cognitive, affective, and social dimensions. In our lessons, students who were initially sport-marginalized did not simply “opt out” of physical activity by taking on non-physical roles. Rather, by acting as creators and analysts, they gained greater ownership over task design and performance criteria, which in turn increased their motivation to attempt shooting movements, to move their bodies, and to experiment with how their bodies responded. The iterative cycles of planning, doing, and reviewing, supported by feedback from peers, teachers, and sensor data, enabled students to experience modest but concrete and meaningful improvements in motor competence.

The case analysis provides concrete, empirically grounded illustrations of how sport-marginalized students engaged with the maker-based PE lessons. In the physical data measurement and performance feedback lessons, students who had previously avoided activities such as flexibility tests, long jump, and shuttle run began to re-enter the learning space when they could work with self-made devices and monitor their own data. For example, Teacher B described how students with low flexibility, who had stayed away from the traditional flexibility test station, repeatedly asked to “try one more time” when using the student-made flexibility meter. Teacher E similarly noted that students were eager to build and test the automatic start sensor and kept challenging their running times once they could see immediate, accurate feedback. In the play- and game-based lessons, Teacher B and Teacher D reported that usually quiet or hesitant students took the lead in using devices they had built and sometimes achieved the highest scores in laser shooting activities. Rather than remaining peripheral to performance-based tasks, these students found new, meaningful ways to participate, which supports the potential of maker-based PE for expanding inclusive participation.

The physical-data measurement and analysis type can promote health awareness and self-management by engaging students in collecting and interpreting their own data using self-made tools. Devices for measuring flexibility, height, and heart rate functioned as health-learning instruments, enabling students to monitor bodily changes and set goals. This extends Ennis [28] concept of health literacy and suggests that tangible, student-made devices can foster ownership and agency over physical wellbeing. The hands-on, constructionist structure may also encourage previously passive learners to view themselves as active managers of their own bodies, linking health and PE within a participatory and student-centered framework.

The performance-feedback and play-based types further strengthened engagement and motivation. Student-built devices automated measurement and provided instant feedback, supporting skill development and self-efficacy [29]. Immediate data visualization encouraged self-regulated learning [30], while cooperative, game-oriented applications such as running games or laser relay games transformed routine measurement into enjoyment and challenge. These activities satisfied autonomy, competence, and relatedness needs as identified by Self-Determination Theory [31] and introduced inclusive forms of gamification using low-cost, student-created technologies.

Overall, Maker-based PE shifts the focus from who participates to how participation is structured and experienced. It repositions marginalized students as health managers, performance analysts, and collaborative innovators. This reframing demonstrates how technology can mediate equity and inclusion in PE [7,32] and offers practical implications for designing participatory, resource-efficient, and technology-integrated lessons that connect maker education with inclusive PE through an embodied, student-driven model of learning.

### 4.2. Shifting Teacher Professionalism Through Maker-Based Pedagogy

The implementation of maker-based PE involved more than adopting new tools; it required a fundamental shift in teachers’ professional orientations and identities. Teachers moved from being transmitters of knowledge to practitioner-experts who continuously adjusted participation structures, diagnosed classroom challenges, and redesigned lessons through experimentation and reflection. This aligns with Avalos [33] and Loughran [34] view of teacher professionalism as a dynamic process shaped by situated practice and ongoing reflection.

A key outcome was the reframing of how teachers perceived sport-marginalized students. Motitswe [35] highlights that teachers’ beliefs and expectations significantly influence their instructional decisions and classroom interactions. In this study, teachers who had viewed students with lower motor skills or confidence as passive began recognizing them as active agents. This change emerged through observing students engaged in device construction, record keeping, and peer feedback. These observations led to redesigned teaching practices that encouraged more inclusive participation.

This transformation extended to collective professional practices. Through collaborative lesson redesign and reflection in a Professional Learning Community (PLC), teachers developed a culture of experimentation and growth. This iterative process reflects Stoll et al. [18] account of innovation institutionalization through shared practice and aligns with Desimone’s principles of effective professional development, including collaboration, reflection, and classroom-based application [36].

Some teachers also chose to explore unfamiliar technological areas, viewing failure as a resource for redesign. This exemplifies teacher agency, which Biesta et al. [37] define as the capacity to act purposefully and reflectively within one’s context. Tao and Gao [38] emphasize that such agency contributes to the reconfiguration of professional identity. In this study, joint problem-solving with students, peer mentoring, and PLC feedback processes supported a collective, practice-based professionalism. The findings suggest that professional growth develops more through co-construction than through isolated expertise.

Alongside these professional shifts, the teachers also identified concrete challenges in implementing maker-based PE. Before the project, several teachers assumed that physical-computing tools would be too difficult or technical for PE specialists, who did not see themselves as “technology teachers.” However, through the PLC workshops they found that basic sensor-based applications could be learned and applied with a relatively small investment of time—often within two to four hours of focused practice. They contrasted this with the months or years they typically devote to mastering and teaching a single sport. In addition, many students already had prior experience with coding and information technology from other subjects, which further lowered the entry threshold for classroom use.

At the same time, the teachers emphasized that the most demanding aspect of Maker Education lay not in learning the tools themselves, but in the workload associated with preparing and managing the hardware. Because the materials were not provided as ready-made kits, they had to purchase components individually, assemble and wire each device, and troubleshoot malfunctions, all of which consumed substantial time and energy. From their perspective, the feasibility of maker-based PE in ordinary schools would be greatly improved if low-cost PE-oriented kits, standardized bills of materials, and basic assembly guides were provided at the school or district level. Such supports would reduce the preparation burden and allow teachers to focus their limited time on pedagogical design and reflection rather than on technical procurement and assembly.

In sum, the professional transformation linked to maker-based PE represents a substantial shift in how teachers understand their roles, enact practice, and approach inclusion. Rather than focusing narrowly on technical competence, this model emphasizes reflective and collaborative professionalism, consistent with recent perspectives on teacher learning as situated and practice-driven. These shifts were documented through PLC meeting records, classroom observations, and teacher interviews, which captured how teachers interpreted and responded to students’ participation in the maker-based lessons.

### 4.3. Practical, Policy, and Global Implications

In summary, maker-based PE extends beyond skill- or competition-centered instruction to create equitable and participatory learning environments. By diversifying student roles, fostering teacher collaboration, and building sustainable school cultures, it demonstrates how technology-integrated pedagogy can promote inclusion and equity in education. Furthermore, the low cost and adaptability of maker-based lessons suggest feasibility even in resource-constrained contexts, aligning with global frameworks such as UNESCO’s Education 2030 Agenda and Sustainable Development Goal 4 (Quality Education). Therefore, maker-based PE can function not only as an instructional model for inclusive practice, but also as a policy-relevant approach that contributes to social inclusion and educational sustainability at both local and global levels. Although context-specific, this study may offer insights for other educational settings with limited resources.

Most of the devices used in the lessons were built from low-cost components, including Arduino-compatible boards, sensors, and basic electronic parts, each typically costing around USD 2 or less. These materials are widely available through local or online suppliers, ensuring accessibility even in resource-constrained school environments.

In addition, the lesson designs, circuit diagrams, and source code have been continuously shared with interested PE teachers through formal in-service workshops and through informal requests via professional networks. These sharing practices have allowed teachers to adapt and implement the activities without requiring highly specialized technical training, supporting the scalability of maker-based PE within ordinary school contexts.

Finally, from a practical standpoint, the study suggests that maker-based PE is more sustainable when teachers manage the workload by beginning with simple, kit-based prototypes, reusing shared lesson resources and code, and receiving structural support from schools or local authorities for procuring and assembling standard sets of components.

## 5. Conclusions

This study demonstrates that a maker-based approach in PE, developed through sustained collaboration within a Professional Learning Community (PLC), can expand participation among sport-marginalized students and support a transformation in teaching practices toward greater inclusivity. Rather than applying preexisting models, the PLC co-designed and refined three types of lessons: physical data measurement and analysis, performance feedback, and play- and game-based activities. These lessons were developed iteratively through collaborative planning, implementation, and reflection, and contributed to diversifying participation by allowing students to engage as performers, makers, operators, and analysts.

This process also supported a reconfiguration of teacher professionalism. Teachers began to view marginalized students as capable and active participants, shifting their pedagogical orientation toward more reflective and student-centered practices. The PLC functioned not only as a design platform but also as a sustained support structure for practitioner-led innovation and shared professional growth.

Furthermore, the affordability and adaptability of maker-based PE suggest its potential suitability for resource-constrained educational contexts. This approach aligns with broader global goals, including UNESCO’s Education 2030 Agenda and Sustainable Development Goal 4 on Quality Education, offering practical and policy-relevant pathways for promoting equity and inclusion in PE.

While this study was grounded in actual classroom experiences, it is best understood as a design-based study incorporating partial implementation. The findings were drawn primarily from teacher-generated data, and future research should incorporate direct student perspectives to more fully understand the impact on sport-marginalized youth.

## 6. Limitations of the Study

This study provides meaningful insights into the inclusive potential of maker-based physical education and the professional growth of teachers; however, several limitations should be noted. First, the research was based on a single Professional Learning Community (PLC) case in South Korea, which may limit the generalizability of the findings due to contextual specificities.

Second, although rich data on student participation were collected through teacher observations, reflective journals, and classroom artifacts, the voices of sport-marginalized students were not obtained through direct interviews or self-reports. Consequently, their experiences and perspectives are represented indirectly through teachers’ interpretations, which may constrain the depth of understanding of their lived experiences.

Third, the researcher’s dual role as both participant and investigator, while beneficial for gaining in-depth contextual insight, involved a potential risk of interpretive bias. To mitigate this concern, strategies such as data triangulation, reflexive journaling, and peer debriefing were employed to enhance the trustworthiness of the analysis.

Future research should adopt mixed-methods designs, include direct accounts from marginalized students, and conduct comparative studies across different institutional and cultural contexts to examine the inclusive impact and practical feasibility of maker-based PE more comprehensively.

## Figures and Tables

**Table 1:** Full coding framework including detailed categories and representative excerpts.

1st Order Concepts	2nd Order Themes	Aggregate Dimensions
Production of measurement devices and data recording Self-monitoring and feedback through time tracking Immediate feedback using LED/buzzer devices Strengthening participation through game-like elements	Real-time Measurement & Feedback Lessons	Maker-based Lesson Types for Enhancing Participation of Sport-marginalized Students (RQ1)
Providing instant feedback through device use Enhancing accuracy and performance through repeated practice Increasing participation through continuous monitoring	Performance Feedback Lessons	
Expanding into games using sensors and buzzers Team relay and cooperative tasks utilizing measurement devices Strengthening engagement through game reward structures	Play & Game-based Lessons	
Recognising diverse roles of marginalized students (makers, operators, data analysts) Reframing marginalized students as active participants with learning potential	Shift in Perceptions of Inclusivity & Student Participation	Teachers’ Reflections on Inclusivity through the Practice of Maker Education (RQ2)
Reflection on traditional skill-centered PE instruction Integration of technical-making with PE instruction Strengthening teachers’ pedagogical adaptability	Professional Reflection and Instructional Reconfiguration	
Joint problem-solving in device making and lesson application Sharing peer experiences and materials Providing psychological support and sustaining a culture of practice-based professionalism	Recognition of the Value of PLCs *	

* PLC = Professional Learning Community.

**Table 2:** Prototypes and their educational implications.

Prototype		Main Function	Application Method	Educational Significance
Physical Data Measurement and Their Educational Implications	Flexibility Measuring Device	Automatic recording of sit-and-reach distance	TOF * sensor + LCD output	Self-regulation through goal-setting and reflective learning experiences
	Height Measuring Device	Automatic calculation of height	Measurement of head-to-floor distance with automated calculation	Self-regulation and development of growth management habits
	Heart Rate Measuring Device	Real-time heart rate monitoring before, during, and after exercise	Pulse sensor + OLED output	Enhanced ability for exercise intensity control, self-regulation, and self-management
Performance Feedback Lessons and Educational Implications	Automatic Timing Start Sensor Device	Automatic detection and recording of start/finish	Enhancing accuracy and fairness in record measurement; providing roles through device operation	Supporting self-regulation; fostering goal-setting and participatory learning experiences
	Shuttle-Run Buzzer	Automatic signal when reaching designated distance	Providing rhythmic cues; granting participation roles and motivation for marginalized students	Promoting self-regulation; fostering growth management habits
	Long-Jump Measuring Device	Automatic measurement of landing distance	Increasing evaluation reliability; enabling self-monitoring and record comparison opportunities	Enhancing understanding of exercise intensity; strengthening self-management competencies
for Performance Feedback Lessons and Educational Implications	Running Games	Automatic recording of start/finish, use of buzzer	Transforming record-keeping into team-based or cooperative running games	Enhancing fairness; expanding participation opportunities for marginalized students
	Cooperative Fitness Games	Use of shuttle-run buzzers and repeated exercise devices	Awarding points for achieving designated repetitions/intensity; cooperative missions	Promoting self-regulation; encouraging sustained participation of students with lower physical ability
	Shooting Relay Games	Use of laser shooting devices, automatic score recording	Relay-format scoring; combining cooperative and competitive elements	Strengthening sense of belonging; providing collaborative learning experiences

* TOF = Time of Flight.

## Data Availability

The data presented in this study are not publicly available due to privacy and ethical restrictions related to sensitive information from participants. However, the data may be made available from the corresponding author upon reasonable request and with permission from the Institutional Review Board.
